# Caution

**Published:** 1875-01

**Authors:** 


CAUTION.

It is distressing to know that our good name is being traded upon by dishonest men. We are constantly in receipt of letters from persons who state that they have paid their money for the JOURNAL OF HEALTH, and have failed to receive it. We have only to say that we promptly supply the JOURNAL to all who pay us for it. We know that irresponsible persons have taken money on our account, but as these individuals have not only acted without authority, but have failed to make returns to us, either of names or money, we could not be expected to mail the JOURNALS. We know, too, that some of our advertising accounts have been fraudulently collected, and we must ask all our patrons to see that receipts are given them for all monies paid, bearing the name of our Treasurer, as none others will be recognized.

The style of our Publishing House changes with the new year. Instead of "E. H. Gibbs & Co.," it is hereafter to be known as THE AMERICAN & FOREIGN PUBLICATION COMPANY. This is not a change in the proprietorship—it is simply a change of form. We now assume the position of an Incorporated Company. This is rendered advisable by our extended business. We are publishing several books, some of which sell mainly in England. We import all periodicals which are published abroad, as they may be ordered. We also receive subscriptions for all American papers and magazines, and guarantee that they will be promptly mailed. We supply books of all kinds at publishers' rates, adding to foreign publications simply the cost of freight and tariff. Our compensation for such service comes solely from the discount which publishers make in consideration of our large orders. The significance of the name of our corporation will be appreciated by all who read this explanation.

The following is a list of our publications:

HALL'S JOURNAL OF HEALTH. Monthly. $2 per year.

BODY AND MIND, a Monthly Health Magazine of great value. $1 per year.

The RED AND GOLD SERIES OF GUIDES, viz.; NEW HOMES; OR, WHERE TO SETTLE, 12mo, cloth binding; 100 pages, with map. Being a full description of the area, population, topography, soil, climate, products, cost of labor, cost of farm animals, price of living, wages of labor &c., in seventeen of the Western States and Territories. To which are added new and valuable articles in relation to cattle and sheep-raising, formation of farms, fruit culture, &c. Price $1. A copy of this elegant book and HALL'S JOURNAL OF HEATH, for one year, will be sent, postage paid, for $2.50.

Appleton's European Guide Book, morocco, gilt edges, 750 pages, 20 maps and 150 engravings. Being a complete guide to England, Ireland, Scotland, France, Germany, Holland, Switzerland, Italy, Austria, Russia, Norway, Denmark and Sweden. It contains complete directions for reaching any point in Europe, the time occupied, and the expense, and the name of all the recommendable hotels. It is the best compendium in existence of the history of Europe, its topography, architecture, works of art, etc. Price, $6. We send this and Hall's Journal of Health, one year, for $6.50.

The Englishman's Illustrated Guide Book to the United States and Canada. A very valuable work of about 280 pages. Price $2. With Hall's Journal, one year, $3.

The New American Guide Book, copiously illustrated with fine steel engravings. Price, $2. With Hall's Journal, one year, $3.

Summer Resorts; containing all needed information concerning the Summer Resorts of the United States; 100 pages; fully illustrated. 50 cents.

Washington and Environs. The best guide-book to the National Capital. Fully illustrated. 50 cents.

